# WT1-pulsed dendritic cell vaccination for neovascular age-related macular degeneration: a phase I pilot feasibility study

**DOI:** 10.1186/s12886-026-04946-y

**Published:** 2026-05-23

**Authors:** Masaki Nakamura, Shigeo Koido, Tuuse Bito, Hiroshi Horiguchi, Yoko Shimizu, Masamori Shimabuku, Junichi Taguchi, Hisato Gunji, Haruo Sugiyama, Tadashi Nakano

**Affiliations:** 1 Department of Ophthalmology, The Jikei University School of Medicine, Tokyo, Japan; 2 Department of Cancer Immunotherapy, The University of Osaka Graduate School of Medicine, Suita, Osaka Japan; 3 Division of Gastroenterology and Hepatology, Department of Internal Medicine, The Jikei University Kashiwa Hospital, Kashiwa, Chiba Japan; 4 Tokyo Midtown Clinic, Minato, Tokyo Japan; 5 Department of Cancer Immunology, The University of Osaka Graduate School of Medicine, Suita, Osaka Japan

**Keywords:** Neovascular age-related macular degeneration, Dendritic cell vaccine, WT1, VEGF, Immunotherapy

## Abstract

**Background:**

Neovascular age-related macular degeneration (nAMD) is a leading cause of vision loss. Although anti-VEGF therapy is effective, frequent injections carry risks and may not fully address the underlying disease process. Wilms’ tumor gene 1 (WT1), an upstream regulator of VEGF, is expressed in choroidal neovascular membranes and may represent a biologically relevant adjunctive target in nAMD. This pilot study evaluated the safety, tolerability, and immunogenicity of WT1-targeted dendritic cell (WT1-DC) vaccines and their effects on retinal morphology and visual function in nAMD patients, with secondary endpoints including clinical outcomes and in vitro WT1-specific T-cell responses.

**Methods:**

Two patients initially received three monthly intravitreal injections of aflibercept (2 mg each), followed by 15 subcutaneous vaccinations with WT1-DCs. The vaccine contained both WT1-specific helper and killer peptides restricted to HLA-A*24:02. In addition, a helper peptide restricted to MHC class II and class I (HLA-A*02:01/02:06) was used.

**Results:**

WT1-DC vaccination was well tolerated, and adverse events were limited. One patient maintained a stable retinal morphology and visual acuity without additional anti-VEGF therapy, whereas the other experienced recurrent subretinal fluid accumulation requiring intermittent rescue injections and a gradual decline in best-corrected visual acuity. Peripheral blood mononuclear cells from both patients exhibited functional CD4 + and CD8 + T-cell responses during vaccination and produced IFN-γ and TNF-α upon WT1 peptide stimulation in vitro.

**Conclusions:**

WT1-DC vaccination was feasible and well tolerated in these two patients who were clinically managed within the nAMD spectrum. Exploratory laboratory findings suggested WT1 peptide–responsive T-cell activity in vitro, but clinically meaningful in vivo immunogenicity was not established. Given the extremely small sample size, the confounding effect of prior anti-VEGF loading therapy, and diagnostic uncertainty regarding CNV etiology in one patient, no conclusions regarding therapeutic efficacy can be drawn. These observations support further investigations of WT1-DC vaccination in larger controlled studies.

**Trial registration:**

Japan Registry of Clinical Trials (jRCTc030210068), registered May 7, 2021.

**Supplementary Information:**

The online version contains supplementary material available at 10.1186/s12886-026-04946-y.

## Background

Age-related macular degeneration is a leading cause of irreversible visual impairment among elderly people worldwide [1]. Neovascular age-related macular degeneration (nAMD) is characterized by pathological choroidal neovascularization (CNV) in the macula, leading to fluid accumulation, hemorrhage, and progressive central vision loss [2]. nAMD is primarily driven by vascular endothelial growth factor (VEGF)-mediated CNV and represents the most common cause of irreversible blindness in developed countries [3, 4]. Since 2006, intravitreal anti-VEGF agents such as ranibizumab, bevacizumab, and aflibercept have revolutionized nAMD management and substantially improved visual outcomes [5–8]. Nevertheless, these therapies do not alter the underlying disease process [9]. Many patients continue to experience vision deterioration, develop treatment resistance, or require frequent injections to maintain disease control [10]. Although treat-and-extend (TAE) and pro re nata (PRN) regimens have been developed to reduce the treatment burden, they still pose economic and logistical challenges for both patients and healthcare systems [11, 12].

Despite the clinical success of anti-VEGF therapy, its limitations are considerable [11, 12]. Repeated injections can cause ocular complications such as macular atrophy and subretinal fibrosis, whereas systemic exposure may lower serum VEGF levels and increase the risks of stroke, myocardial infarction, and thromboembolic events [13–15]. Moreover, the long-term durability of treatment is limited; the Seven-Up Study revealed that initial visual gains were not maintained after seven years [16, 17]. Patient adherence also remains a challenge, as fear of and discomfort from injections, coupled with the transient nature of treatment effects, reduce long-term compliance. The persistence of disease activity despite VEGF inhibition highlights the involvement of additional molecular pathways and disease-associated antigens, underscoring the need for novel therapeutic strategies beyond the VEGF-centered paradigm. Together, these limitations emphasize the urgent need for alternative therapeutic approaches. In particular, novel strategies for nAMD that can achieve durable efficacy, minimize adverse effects, and improve patient adherence are urgently needed.

Wilms’ tumor gene 1 (WT1) is a zinc finger transcription factor that is aberrantly overexpressed in various malignancies, where it functions as a tumor-associated antigen [18]. Because of its restricted expression in normal adult tissues and strong immunogenicity, WT1 has been widely studied as a target for cancer immunotherapy [19]. Importantly, WT1 has attracted attention in ophthalmology because it is highly expressed in the CNV membranes of patients with nAMD and in murine models, particularly within vascular endothelial cells [20]. Evidence suggests that WT1 may act upstream of VEGF to drive pathological angiogenesis, indicating that WT1 represents not only a disease-associated antigen in ocular angiogenesis but also a potential regulatory factor in CNV development [18].

Preclinical studies have shown that immunotherapeutic approaches can suppress ocular neovascularization; moreover, WT1 is a key regulator of pathological angiogenesis, supporting the rationale for using WT1-targeted strategies to treat the eye [18, 21, 22]. In a murine model, vaccination with WT1-derived killer peptide (KP) and helper peptide (HP) induced robust WT1-specific cytotoxic T lymphocytes (CTLs) that infiltrated tumors and exerted antitumor effects [23]. These findings provide a mechanistic rationale for exploring similar WT1-targeted immunotherapeutic approaches in ocular neovascularization models. Dendritic cells (DCs), which are professional antigen-presenting cells, play a central role in regulating immune responses [24]. WT1 peptide-pulsed DCs can elicit robust WT1-specific immunity, including CD4 + helper T cells and CD8 + CTLs [19]. A novel WT1-derived HP (WT1-HP) further enhances the activation of WT1-specific Th1 cells and CTLs [25, 26]. In addition, prior clinical experience with WT1-DC vaccination in pancreatic cancer supports the feasibility of this platform in humans [27]. Because WT1 is expressed in CNV membranes and may be involved in VEGF-related angiogenic signaling, targeting WT1 could represent a biologically relevant adjunctive approach to existing anti-VEGF therapy in nAMD. On the basis of this rationale, we conducted this first-in-human exploratory phase I pilot study to evaluate the feasibility, safety, and exploratory immunologic findings of WT1-DC vaccination in patients with nAMD.

## Methods

### Study design

This study was a noncomparative, open-label, phase I study (trial registration number jRCTc030210068). As this study represents a first-in-human trial in patients with nAMD, the primary endpoints were safety and toxicity according to the Common Terminology Criteria for Adverse Events (CTCAE; V.5.0) (available at https://ctep.cancer.gov/protocoldevelopment/electronic_applications/ctc.htm). The secondary endpoints included the mean change in best corrected visual acuity (BCVA) from baseline, the change in central retinal thickness (CRT) measured by spectral-domain optical coherence tomography (OCT) and the change in CNV lesion area evaluated by OCT angiography, both of which were acquired on the Cirrus HD-OCT 4000 with AngioPlex (Carl Zeiss Meditec, Dublin, CA, USA). In addition, WT1-specific immune responses, assessed by in vitro experiments, were evaluated as an exploratory secondary endpoint. Owing to the exploratory, open-label design, neither the treating physicians nor the outcome assessors were masked to the treatment.

### Patient population

nAMD is diagnosed on the basis of clinical findings and imaging. OCT is the primary modality for detecting subretinal fluid (SRF) or intraretinal fluid (IRF), and OCT angiography can be used to delineate the macular neovascular complex. Moreover, HLA typing, including HLA-A, -DR, -DP, and -DQ loci, was performed in patients with nAMD to assess eligibility for WT1-DC vaccination. Additionally, patients with adequate hematologic and organ function were included. The exclusion criteria were serious infections, severe underlying diseases, severe allergic disease, and a history of receiving antitumor therapy within the past year. All patients provided written informed consent. Patients with nAMD were screened consecutively without restriction by nAMD subtype.

### WT1-DC vaccines

Peripheral blood mononuclear cell (PBMC)-rich fractions were collected using leukapheresis with a Spectra Optia^®^ cell separator (Terumo BCT, Inc., Tokyo, Japan). Adherent PBMCs were cultured with 50 ng/mL GM-CSF (Gentaur, Brussels, Belgium) and 50 ng/mL IL-4 (R&D Systems, Inc., Minneapolis, MN, USA) for 5 days to generate immature DCs. Immature DCs were subsequently cultured with 10 µg/mL lyophilized *Streptococcus pyogenes* of low virulence (OK-432; Chugai Pharmaceutical, Tokyo, Japan) and 50 ng/mL prostaglandin E2 (PGE2) (Daiichi Fine Chemical Co., Ltd., Toyama, Japan) supplemented with 20 µg/mL of a novel WT1 peptide cocktail for 24 h to generate WT1-DCs. The novel WT1 peptide cocktail included WT1-HP (amino acids 34–51: WAPVLDFAPPGASAYGSL), which is presented by MHC class II and HLA-A*02:01 or HLA-A*02:06, and WT1-24:02-KP (amino acids 235–243: CYTWNQMNL), as previously described [25]. WT1-DCs were incubated with the following monoclonal antibodies (mAbs) to assess their phenotypes: fluorescein isothiocyanate (FITC)-conjugated anti-human CD14 (61D3) (Thermo Fisher Scientific, Waltham, Massachusetts, USA), HLA-ABC (W6/32), CD80 (2D10), CD40 (5C3), phycoerythrin (PE)-conjugated anti-human CCR7 (150503) (all from R&D Systems, Minneapolis, Minnesota, USA), CD11c (3.9), HLA-DR (L243), CD83 (HB 15e), and CD86 (IT2.2) (all from BioLegend, San Diego, California, USA). Moreover, PBMCs, immature DCs, and WT1-DCs were counted via trypan blue exclusion to assess their viability and recovery rates. Each dose of the WT1-DC vaccine contained approximately 1 × 10⁷ DCs suspended in 0.5 mL of saline supplemented with OK-432. The dose of OK-432 was increased in a stepwise manner: 0.2 Klinische Einheit (KE) for the initial dose, 0.5 KE for the second, and 1.0 KE for all subsequent administrations. The vaccine was administered subcutaneously at six anatomical sites (bilateral upper arms, lower abdomen, and femoral regions) at intervals of 2 to 4 weeks. Each patient received up to 15 doses, as a single leukapheresis procedure usually provided sufficient DCs to prepare at least 15 vaccinations.

### Treatment protocol

Patients with nAMD received a loading phase of intravitreal aflibercept (2 mg/0.05 mL; Bayer Yakuhin, Ltd., Osaka, Japan), which was administered once monthly for three consecutive doses, to inhibit VEGF signaling and associated downstream processes. WT1-DC vaccines were administered four weeks after the final aflibercept (2 mg) injection. In patient AMD-WT1-01, the first six vaccinations were administered at two-week intervals, after which the interval was extended to four weeks, totaling fifteen doses. In contrast, patient AMD-WT1-02 received all 15 vaccinations at two-week intervals.

### Disease activity monitoring and rescue therapy

Disease activity was assessed three times during the WT1-DC treatment phase and throughout the 6-month post-treatment observation period. In the original study protocol, rescue intravitreal aflibercept (2 mg) was prespecified for standardized monitoring when any of the following findings were present: (1) an increase in CRT of ≥ 100 μm from the lowest recorded value, (2) the appearance of a new CNV lesion on OCT angiography, or (3) the appearance of new macular hemorrhage on color fundus photography. However, in the two cases reported here, retreatment decisions were not determined solely by these protocol-defined thresholds. Instead, retreatment was based on the overall recurrence or worsening of exudative activity on multimodal imaging, particularly subretinal or intraretinal fluid on OCT, together with clinical judgment. Best-corrected visual acuity was not used as an independent retreatment criterion.

### Evaluation of adverse events and clinical outcomes

Adverse events, including fever and injection site reactions, were monitored and graded according to CTCAE (V.5.0). These events were assessed after each vaccination in all patients. Injection site reactions were evaluated by patient self-assessment at 48 h post-injection. Ocular adverse events were evaluated using clinically relevant criteria. Clinical outcomes were evaluated by BCVA measured with standard Landolt-C charts and recorded in decimal notation. Morphological assessment was performed using fundus photography, OCT and OCT angiography.

### WT1 peptide-specific delayed-type hypersensitivity (WT1-DTH) test

The WT1-DTH test was conducted once before treatment and at each vaccination time point. Briefly, 20 µg of either the WT1 peptide (WT1-24:02-KP or WT1-HP) dissolved in saline or saline alone as a control was injected subcutaneously into the forearm. The diameter of the resulting erythema was measured 48 h after the injection. WT1-DTH positivity was defined as erythema greater than 2 mm in diameter.

### Analysis of WT1-specific immune responses

To assess the induction of WT1-specific immune responses via WT1-DC vaccines, PBMCs (5 × 10^5^/mL) were stimulated with 10 µg/mL HLA-A-matched WT1-derived killer peptide (WT1-KP) and 20 µg/mL WT1-HP (both from Greiner Bio-One GmbH, Frickenhausen, Germany) in the presence of 10 U/mL rhIL-2 (Shionogi & Co., Osaka, Japan) and 10 ng/mL IL-7 (PeproTech, Rocky Hill, New Jersey, USA) for 9 days. HLA-A type-matched WT1-KPs were selected as follows: HLA-A*02:06-restricted WT1-KP (amino acids 37–47: VLDFAPPGASA, 37–47) for patient AMD-WT1-01 or WT1-24:02-KP (amino acids 235–243: CYTWNQMNL) for patient AMD-WT1-02. HIV Gag p17 peptides restricted for HLA-A*02:06 (amino acids 77–85: ILKEPVHGV) (GenScript Biotech Corporation, Tokyo, Japan) or HIV Env (gp160) peptide restricted for HLA-A*24:02 (amino acids 584–592 RYLRDQQLL) (BioLegend) and matched isotype immunoglobulin G (IgG) (BioLegend) were used as negative controls. The cells were restimulated with 10 µg/mL HLA-A type-matched WT1-KP, 20 µg/mL WT1-HP, and 10 µg/mL brefeldin A (Sigma–Aldrich; Merck KGaA, Darmstadt, Germany) for 5 hours. The cells were stained with the following mAbs: FITC-conjugated anti-human CD8 (T8) (Beckman Coulter, Brea, California, USA), APC-Cy7-conjugated anti-human CD4 (OKT4), APC-conjugated anti-human IFN-γ (B27), or APC-conjugated anti-human TNF-α (MAb11) (all from BioLegend). IFN-γ-producing or TNF-α-producing CD4 + or CD8 + T cells were analyzed. Moreover, patient AMD-WT1-02 with an HLA-A*24:02 allele was evaluated for the induction of HLA-A*24:02-restricted WT1-CTLs producing IFN-γ or TNF-α via PE-conjugated anti-human HLA-A*24:02-modified WT1-tetramer amino acids 235–243: CYTWNQMNL (MBL, Nagoya, Japan). PE-conjugated anti-human HLA-A*24:02-HIV (env gp160)-tetramer amino acids 584–592: RYLRDQQLL (MBL) was used as a negative control.

### Flow cytometry analysis

The phenotypes of the cells were analyzed using an Attune NxT Flow Cytometer (Thermo Fisher Scientific) and FlowJo analysis software V.10.10.0. (Tree Star, Ashland, Oregon, USA).

## Results

### Patient characteristics

Four patients with nAMD were screened for eligibility. Two patients were enrolled in this pilot study, whereas two eligible patients declined participation. No patients were excluded on the basis of predefined exclusion criteria. Because recruitment was not restricted by the nAMD subtype, the two enrolled patients represented different clinical subtypes of nAMD. As shown in Table 1, both enrolled patients were female. Patient AMD-WT1-01 was 70–79 years old, with involvement of the left eye, and was diagnosed with type 1 macular neovascularization (MNV) of the polypoidal choroidal vasculopathy (PCV) subtype. The baseline decimal BCVA was 0.6, and the spherical equivalent was + 1.5 diopters. OCT demonstrated SRF in association with polypoidal lesions, and en face OCT angiography of the outer retina to the choriocapillaris (ORCC) slab revealed a branching vascular network (BVN). She also had cataracts but no systemic comorbidities. Her HLA typing revealed HLA-A*02:06/11:01, HLA-DRB1*12:01/14:06, HLA-DQB1*03:01, and HLA-DPB1*02:01/05:01. Patient AMD-WT1-02 was 80–89 years old, with involvement of the right eye, and was clinically managed as having type 1 MNV within the nAMD spectrum. The baseline BCVA was 0.7, the spherical equivalent was − 6.5 diopters, and the axial length was 27.9 mm. OCT revealed a homogeneous hyperreflective subretinal lesion beneath the fovea, which was consistent with submacular hemorrhage. En face OCT angiography of the ORCC slab did not clearly delineate the MNV. No other ocular comorbidities were present. Her medical history revealed that she had been cured of breast cancer. Given the high degree of myopia and long axial length, however, the CNV etiology in this case remained clinically uncertain. HLA typing revealed HLA-A*24:02/31:01, HLA-DRB1*15:01/15:02, HLA-DQB1*06:01/06:02, and HLA-DPB1*05:01/09:01.

**Table 1:** Baseline characteristics of the patients

Variables	AMD-WT1-01	AMD-WT1-02
Age (years)	70–79	80–89
Sex	Female	Female
Affected eye	Left	Right
Diagnosis	Type 1 MNV (PCV subtype)	Type 1 MNV*
Spherical equivalent (D)	+ 1.5	−6.5
Baseline decimal BCVA	0.6	0.7
Axial length (mm)	Not measured	27.9
Baseline OCT findings	Subretinal fluid	Macular hemorrhage
Other ocular comorbidities	Cataract	None
Systemic comorbidities	None	Breast cancer (cured)
HLA typing	HLA-A*02:06/11:01, HLA-DRB1*12:01/14:06, HLA-DQB1*03:01, HLA-DPB1*02:01/05:01	HLA-A*24:02/31:01, HLA-DRB1*15:01/15:02, HLA-DQB1*06:01/06:02, HLA-DPB1*05:01/09:01

* In patient AMD-WT1-02, the CNV etiology remained clinically uncertain. The spherical equivalent was − 6.5 diopters, and the axial length was 27.9 mm

AMD: age-related macular degeneration, WT1: Wilms’ tumor 1, DC: dendritic cell, MNV: macular neovascularization, PCV: polypoidal choroidal vasculopathy, BCVA: best corrected visual acuity, OCT: optical coherence tomography, HLA: human leukocyte antigen

### Phenotype of WT1-DCs

**Fig. 1:**
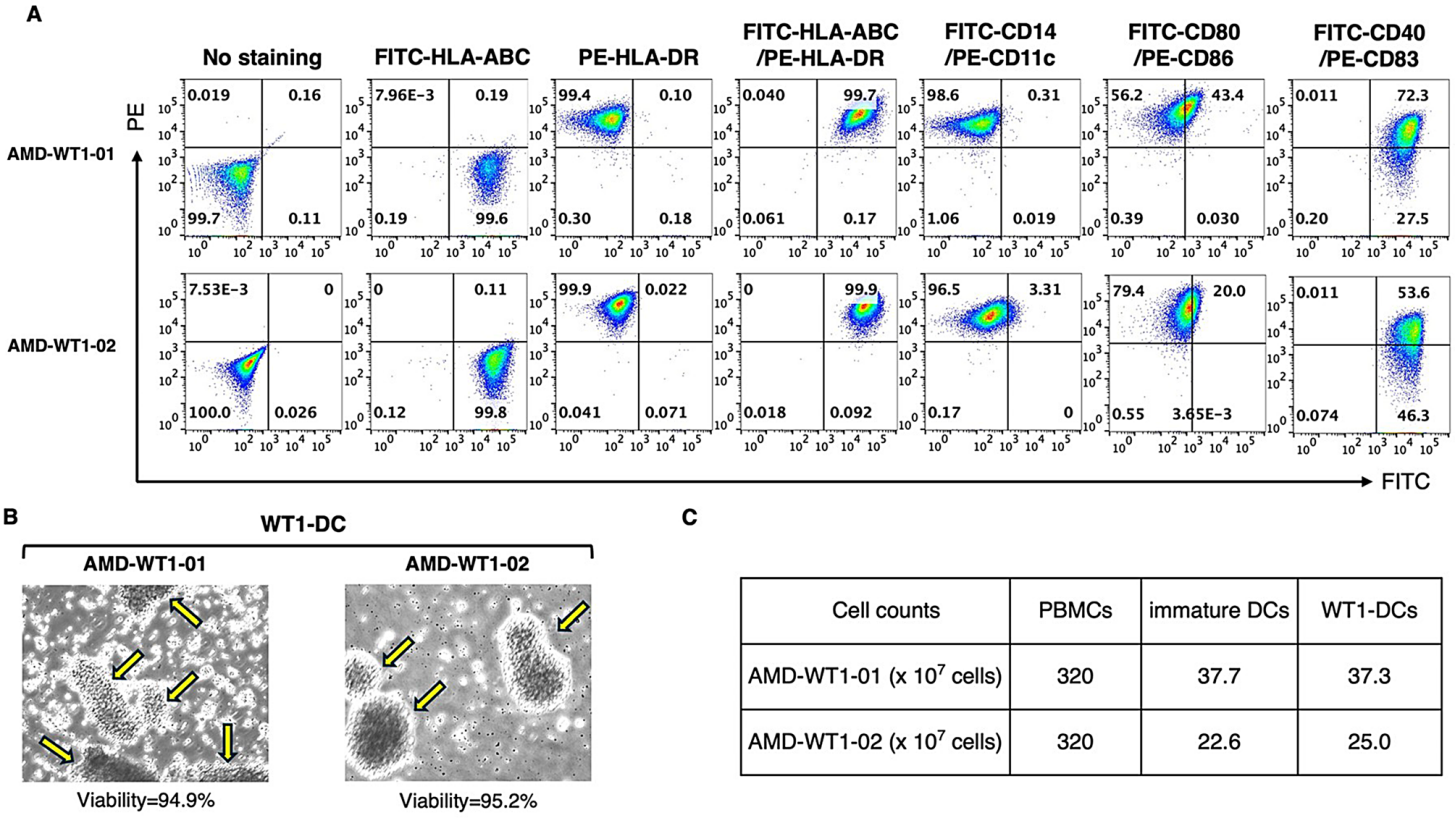
Phenotype and characteristics of WT1-DCs. **A**, Expression of the indicated molecules (HLA-ABC, HLA-DR, CD14, CD11c, CD80, CD86, CD40, and CD83) on WT1-DCs from two patients with nAMD (AMD-WT1-01 and AMD-WT1-02). Numbers indicate events in each gated region. **B**, Morphological features of WT1-DCs, showing dendrite and cluster formation (yellow arrows) in both patients, as observed under an inverted microscope (× 40). **C**, Cell counts (×10⁷) of PBMCs, immature DCs, and WT1-DCs. PBMC, peripheral blood mononuclear cell; DC, dendritic cell; WT1-DC, WT1-pulsed dendritic cell

WT1-DCs presented high levels of HLA-ABC, HLA-DR, CD86, CD40, and CD11c; moderate levels of CD80 and CD83; and low levels of CD14 (Fig. 1A). DCs formed cells that were clustered during culture with WT1 peptides (Fig. 1B). The recovery rates of WT1-DCs from immature DCs from patients AMD-WT1-01 and AMD-WT1-02 were 98.9% and 111%, respectively (Fig. 1C).

### Toxicity

No adverse events related to intravitreal aflibercept injections were observed in either patient. Both exhibited mild (CTCAE grade 1) injection site reactions associated with WT1-DC vaccination, and neither experienced any other adverse events, including fever. All patients successfully completed 15 doses of WT1-DC vaccination without experiencing any vaccine-related toxicity.

### Clinical course and treatment outcomes

Two patients with nAMD received WT1-DC vaccination following a standard loading phase of three monthly intravitreal 2 mg aflibercept injections. WT1-DC vaccination was initiated at week 14 in patient AMD-WT1-01 because of a scheduling issue, whereas it was initiated at week 12 in patient AMD-WT1-02. Patient AMD-WT1-01 completed 15 WT1-DC doses by week 64 and needed three additional rescue aflibercept injections (2 mg) at weeks 28, 52, and 72 because of recurrent SRF. In contrast, patient AMD-WT1-02 completed 15 doses by week 40 and remained stable without rescue therapy or the recurrence of exudative lesions throughout follow-up.

Following the aflibercept loading phase, the BCVA was 0.6 for patient AMD-WT1-01 and 0.8 for patient AMD-WT1-02. At the last visit, the BCVA was 0.4 and 0.7, respectively. The detailed clinical course of each patient is summarized in Table 2 to further illustrate the individual treatment responses.

**Fig. 2:**
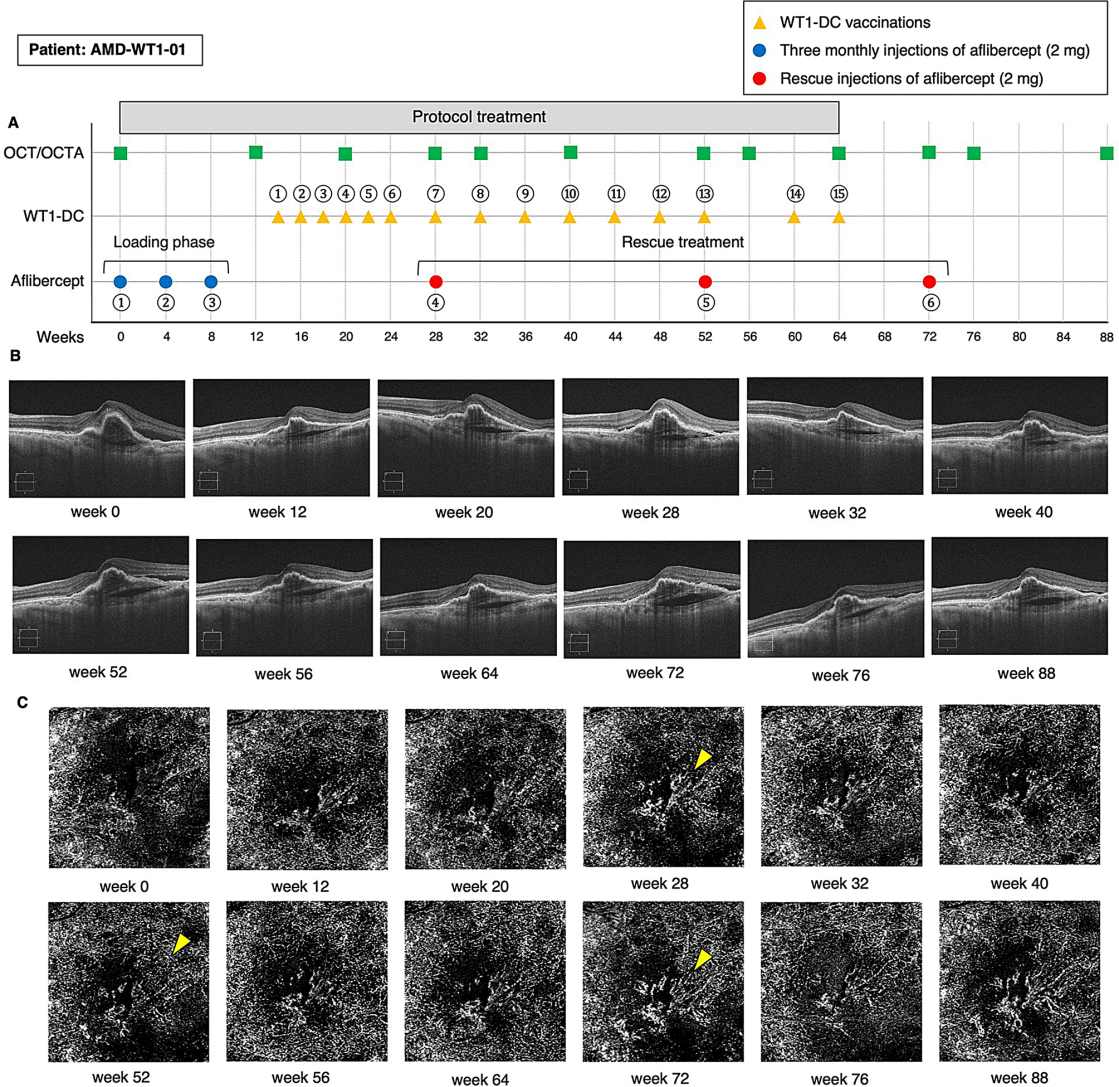
Clinical course, OCT B-scans, and en face OCTA findings for patient AMD-WT1-01. **A**, Timeline of treatment and evaluation over 88 weeks. The blue circles indicate three monthly injections of aflibercept (2 mg) during the loading phase (weeks 0, 4, and 8). Yellow triangles denote WT1-DC vaccinations, which were administered every 2 weeks for the first six doses and every 4 weeks thereafter up to dose 15. The green squares indicate OCT/OCTA assessment visits. Red circles represent rescue injections of 2 mg of aflibercept that were administered for recurrent exudative activity on follow-up multimodal imaging. **B**, Representative horizontal foveal OCT B-scans at 12 visits (weeks 0, 12, 20, 28, 32, 40, 52, 56, 64, 72,76, and 88). Subretinal fluid (SRF) resolved after the loading phase, recurred at weeks 28, 52, and 72, and regressed following rescue injections. **C**, En face OCT angiography of the outer retina to the choriocapillaris (ORCC) slab displayed at the same time points as in panel B (weeks 0, 12, 20, 28, 32, 40, 52, 56, 64, 72,76, and 88). The macular neovascular complex shows a branching vascular network (BVN) that becomes more extensive at visits with recurrent SRF, with peripheral arborization (arrowheads)

The treatment timeline, representative foveal OCT B-scans, and time-matched en face OCT angiography of the ORCC slab of patient AMD-WT1-01 are shown in Fig. 2A–C.

**Fig. 3:**
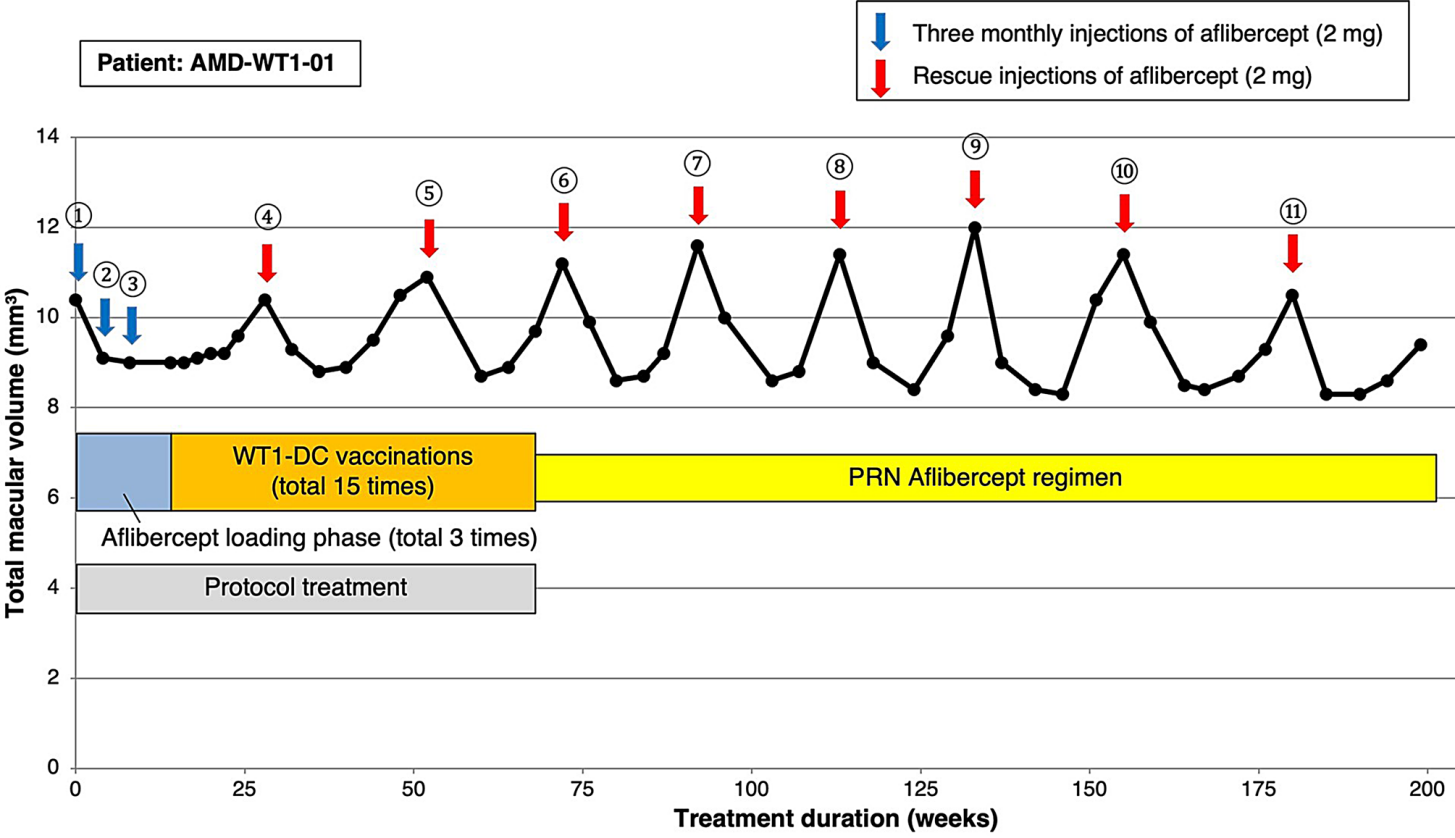
Longitudinal treatment course of total macular volume (TMV) in patient AMD-WT1-01. TMV (mm³) is plotted over time from baseline. After the initial loading phase of three monthly intravitreal aflibercept injections (2 mg; blue downward arrows), WT1-DCs were administered 15 times during the protocol treatment period. Thereafter, the patient was managed with a pro re nata (PRN) aflibercept regimen, during which a total of eight additional aflibercept injections (2 mg; red downward arrows) were administered up to the most recent follow-up

After the resolution of the SRF with the aflibercept loading phase, WT1-DC vaccination was initiated and completed with 15 doses by week 64. SRF recurred at weeks 28, 52, and 72, with each episode prompting a rescue aflibercept injection. In this patient, retreatment was based on recurrent exudative activity on follow-up multimodal imaging, particularly for SRF on OCT, rather than on protocol-defined CRT thresholds alone. Thereafter, a small amount of residual SRF persisted, and the patient was maintained on a PRN aflibercept regimen. As of 135 weeks after the completion of the vaccination, the patient had received a total of 11 aflibercept injections under the PRN regimen. The longitudinal treatment course from baseline to the most recent visit is summarized in Fig. 3.

**Fig. 4:**
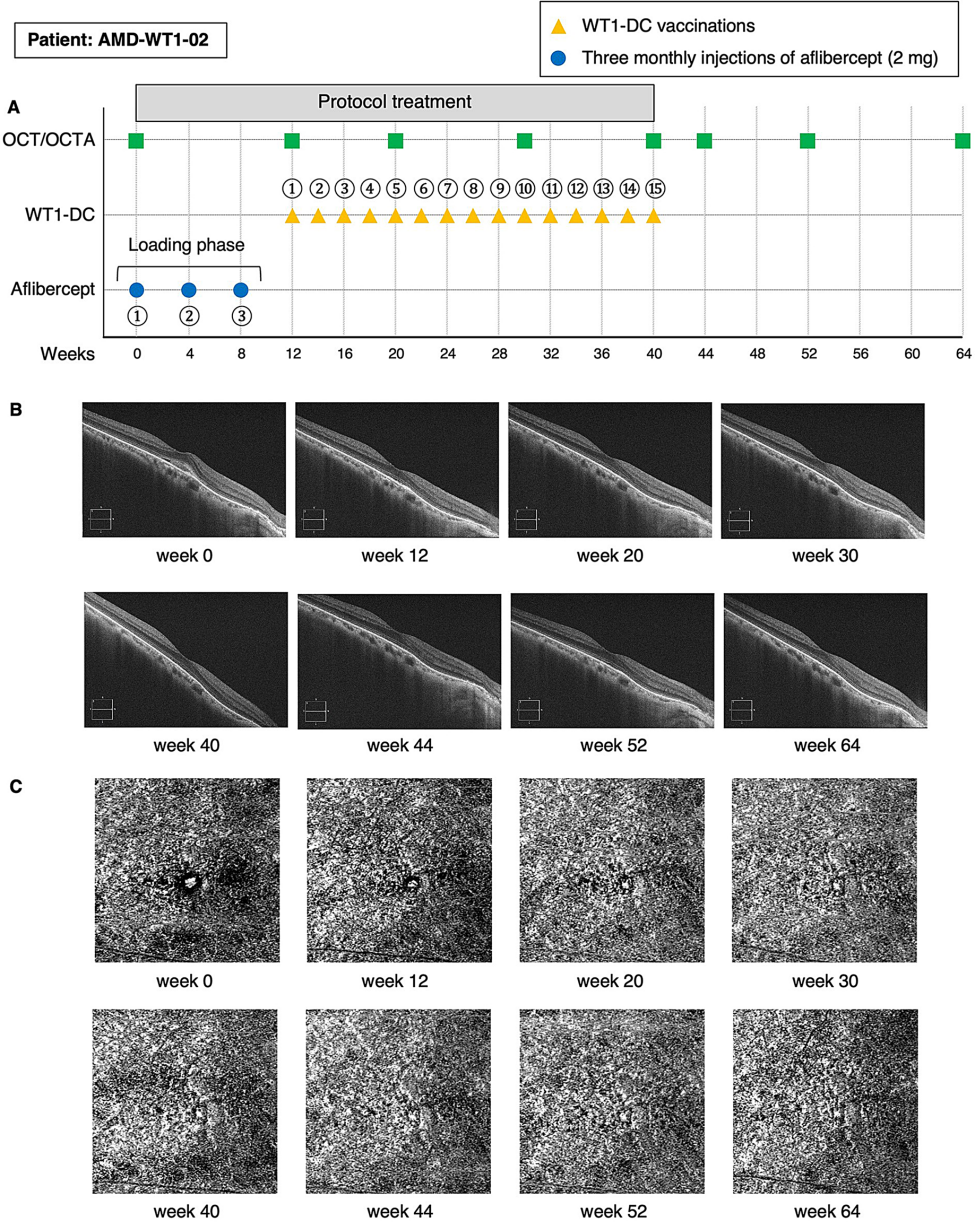
Clinical course, OCT B-scans, and en face OCTA findings for patient AMD-WT1-02. **A**, Timeline of treatment and evaluation over 64 weeks. The blue circles indicate three monthly injections of aflibercept (2 mg) during the loading phase (weeks 0, 4, and 8). Yellow triangles denote WT1-DC vaccinations administered every 2 weeks for all 15 doses. The green squares indicate OCT/OCTA assessment visits. No rescue aflibercept injections were required during treatment or follow-up. **B**, Representative horizontal foveal OCT B-scans at eight visits (weeks 0, 12, 20, 30, 40, 44, 52, and 64) showing the resolution of macular hemorrhage and the absence of fluid throughout the observation period. **C**, En face OCT angiography (ORCC slab) displayed at the same time points as in panel B (weeks 0, 12, 20, 30, 40, 44, 52, and 64). The macular neovascular complex remains indistinct at baseline and shows no appreciable interval change during or after vaccination, which is consistent with the absence of exudation

The clinical course with corresponding OCT B-scans and ORCC en face OCTA of patient AMD-WT1-02 is summarized in Fig. 4A–C.

Vaccination was initiated after the resolution of macular hemorrhage; throughout vaccination and follow-up, neither fluid nor hemorrhage recurred, no rescue injections were needed, and the BCVA remained stable without additional treatment for at least 104 weeks after the completion of vaccination.

WT1-DC vaccination was administered without vaccine-related toxicity in either patient. Given the uncontrolled design, prior anti-VEGF loading therapy, and marked case heterogeneity, the subsequent clinical observations should be interpreted as descriptive rather than as evidence of therapeutic efficacy.

**Table 2:** Clinical course and treatment outcomes of two patients clinically managed within the nAMD spectrum who received WT1-DC vaccination

Variables	AMD-WT1-01	AMD-WT1-02
WT1-DC initiation (week)	14	12
WT1-DC completion (week)	64	40
Total number of WT1-DC injections	15	15
Rescue aflibercept injections	3 (weeks 28, 52, and 72)	0
Disease activity	SRF recurrence (3 episodes)	None
Decimal BCVA after loading phase	0.6	0.8
Decimal BCVA after WT1-DC	0.4 (week 88)	0.6 (week 64)
Final decimal BCVA (end of follow-up)	0.4	0.7
Post-treatment status	PRN regimen ongoing	Stable without additional therapy

nAMD: neovascular age-related macular degeneration, WT1: Wilms’ tumor 1, DC: dendritic cell, BCVA: best corrected visual acuity, SRF: subretinal fluid, PRN: pro re nata

### WT1-specific immunity

**Fig. 5:**
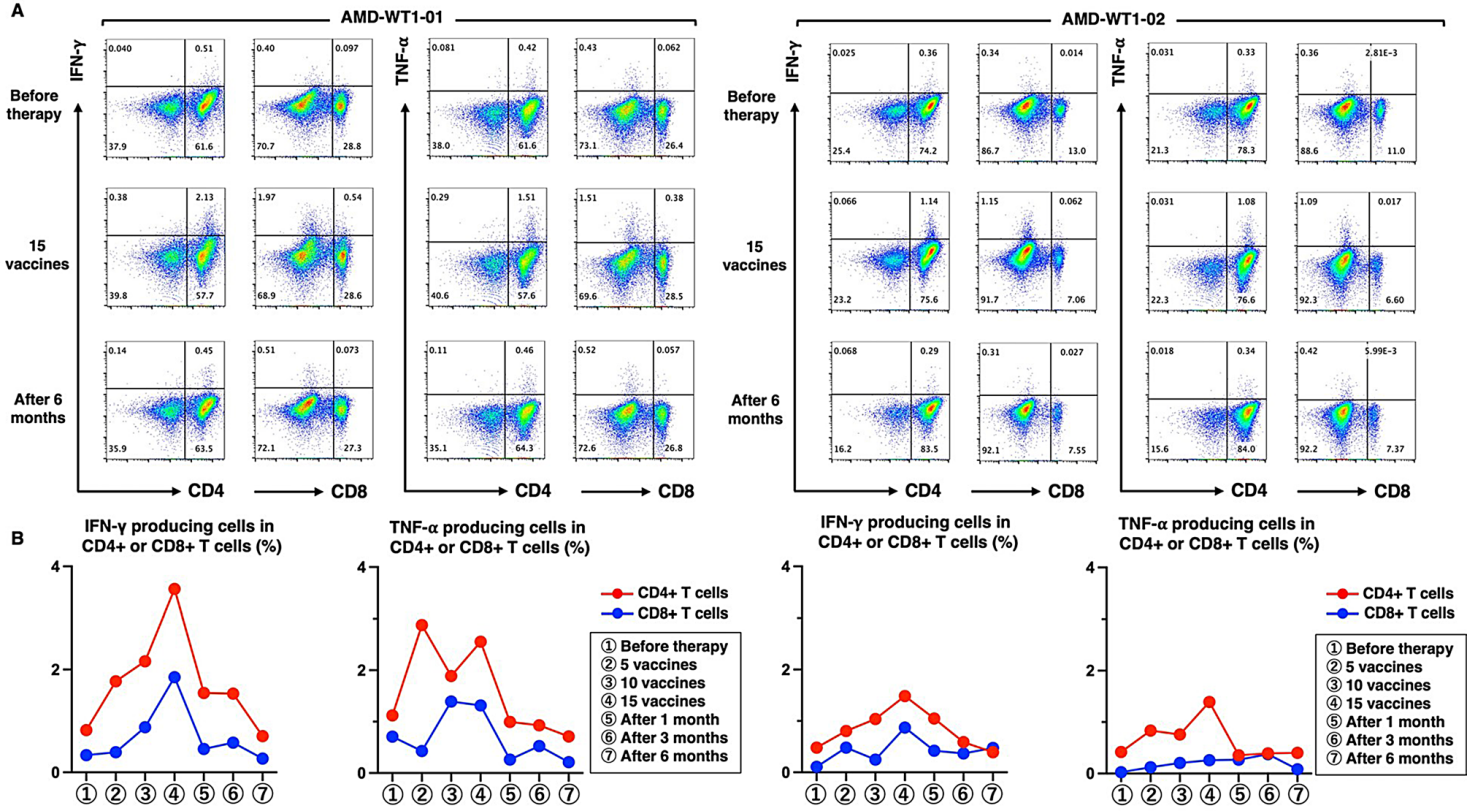
Induction of WT1-specific immunity in patients with nAMD. **A**, Production of IFN-γ (left panel) or TNF-α (right panel) by CD4 + and CD8 + T cells in patients with nAMD (left panel: patient AMD-WT1-01; right panel: patient AMD-WT1-02) at predefined time points: before therapy; after the 5th, 10th, and 15th vaccinations; and at 1, 3, and 6 months after the final vaccination. **B**, Proportions of IFN-γ (left panel) or TNF-α (right panel)-producing CD4 + and CD8 + T cells among total CD4 + and CD8 + T cells in patients with nAMD (left panel: patient AMD-WT1-01; right panel: patient AMD-WT1-02) at predetermined time points: prior to initiation of therapy; following the 5th, 10th, and 15th vaccinations; and at 1, 3, and 6 months after completion of the 15th vaccination

Both patients exhibited negative DTH responses to WT1 peptides (WT1-KP or WT1-HP) prior to treatment and throughout the vaccination period. Therefore, to further evaluate WT1-specific immunity in greater detail, PBMCs were stimulated in vitro with HLA-A type-matched WT1-KP and WT1-HP according to each patient’s HLA-A type. The induction of WT1-specific IFN-γ and TNF-α production by CD4 + or CD8 + T cells was subsequently assessed. As shown in Fig. 5A, IFN-γ and TNF-α production was measured in both CD4 + T cells and CD8 + T cells from each patient upon stimulation with a novel WT1 peptide cocktail in vitro. The production of IFN-γ and TNF-α by both CD4 + and CD8 + T cells increased during WT1-DC vaccination in both patients. However, the levels of these cytokines in the two patients reverted to baseline levels within one month after the completion of the vaccination series (Fig. 5B).

The induction of HLA-A*24:02-restricted WT1-CTLs was analyzed in detail using HLA-A*24:02-restricted WT1 tetramers. Patient AMD-WT1-02, who carried this allele, was the focus of this analysis, as suitable tetramers for other HLA-A alleles were not available [27]. Under the experimental conditions, HLA-A*24:02-restricted WT1-CTLs were not detected during therapy (Additional file 1).

## Discussion

The primary endpoints were defined as the safety and toxicity of WT1-DC vaccination in patients with nAMD. Secondary endpoints were defined as clinical outcomes associated with WT1-DC administration and the induction of WT1-specific immune responses in this pilot study.

In patients with nAMD, tissue biopsy is not feasible for assessing neovascular lesions, making direct evaluation of WT1 expression particularly challenging. This limitation complicates the identification of individuals most likely to benefit from WT1-targeted immunotherapy. Nevertheless, WT1 is highly expressed in CNV membranes, especially within vascular endothelial cells, where it has been implicated in neovascularization [18, 20, 28]. However, in the present two-patient, uncontrolled pilot study, no conclusion can be drawn regarding whether WT1-DC vaccination altered neovascular activity, reduced exudative recurrence, or decreased the anti-VEGF treatment burden. At present, clinical observations should be regarded as only preliminary and hypothesis-generating.

The relevance of the HLA-restricted WT1 peptides used in this study must also be considered in the context of HLA typing. The efficacy of WT1-DC vaccination likely depends on the compatibility between the administered peptides and the patient’s HLA alleles, as peptide presentation by specific HLA molecules is critical for eliciting a robust WT1-specific T-cell response. Accordingly, evaluating HLA compatibility is essential for selecting patients most likely to benefit from WT1-targeted vaccination strategies in nAMD. Patient AMD-WT1-01 expressed HLA-A*02:06. This allele restricts WT1-HP presented by HLA-A*02:06 [25]. In contrast, patient AMD-WT1-02 expressed HLA-A*24:02, which restricts WT1-24:02-KP. In addition, both patients expressed MHC class II molecules capable of presenting WT1-HP [25, 26]. Therefore, both patients were eligible to receive both WT1-HP and WT1-24:02-KP. In this study, a peptide cocktail comprising WT1-HP and WT1-24:02-KP was employed, supporting the rationale that WT1-DC vaccination represents a feasible approach to induce WT1-specific immunity in enrolled patients with nAMD. This treatment strategy is indeed dependent on HLA typing, which may raise concerns regarding its generalizability and clinical applicability. Given that approximately 80% of Japanese individuals carry at least one HLA-A24:02, HLA-A02:01, or HLA-A*02:06, this strategy may be applicable to a large proportion of the population [29]. Therefore, a large proportion of Japanese patients can be covered by targeting these alleles. The class II peptides used in this study contain multiple epitopes, including those restricted by HLA-A*02:01 and HLA-A*02:06, enabling broader applicability across different HLA types. Moreover, even in cases where HLA class I is not matched, the WT1 helper peptide employed in this study is capable of binding to a wide range of HLA class II molecules, allowing its use in virtually all patients. Importantly, WT1-specific CD4 + T cells with cytotoxic activity have been reported to be inducible [30]. Taken together, these considerations suggest that the WT1 peptide cocktail used in this study may have broader applicability than a single HLA-restricted peptide does. However, the clinical applicability of this strategy remains dependent on the HLA background and should be interpreted in a population-specific context.

The safety profile, which was defined as the primary endpoint of this study, further supports the feasibility of WT1-DC vaccination in patients with nAMD. Neither patient experienced adverse events related to intravitreal aflibercept injections, and both tolerated the vaccination protocol without difficulty. Injection site reactions associated with WT1-DC were limited to mild, transient events (CTCAE grade 1), and no systemic adverse events such as fever were observed. Importantly, both patients successfully completed all 15 doses of WT1-DC therapy without interruption, and no vaccine-related toxicity was detected. These findings indicate that WT1-DC vaccination was well tolerated in this limited pilot experience. Nonetheless, given the small sample size and limited follow-up period, larger controlled studies with extended follow-up periods will be necessary to confirm the long-term safety of this approach.

The favorable safety profile of WT1-DC vaccination is particularly noteworthy when it is considered in the context of current anti-VEGF therapies. Although intravitreal anti-VEGF injections are generally regarded as safe, they carry a small but well-recognized risk of ocular complications, including endophthalmitis, intraocular inflammation, and retinal detachment, as well as systemic risks associated with VEGF suppression, such as cardiovascular and thromboembolic events [31, 32]. The intravitreal pharmacokinetics of anti-VEGF agents depend on the vitreous status [33]. In contrast, WT1-DC vaccination was not associated with adverse ocular or systemic effects [19]. Therefore, this subcutaneous immunotherapeutic approach does not contribute to the established risks of anti-VEGF therapy, avoiding vitreous-dependent risks. From the perspective of cancer immunotherapy, where DC-based vaccines have been extensively evaluated, adverse events such as fever, malaise, and injection site inflammation are relatively common but generally manageable. The absence of systemic immune-related toxicity in these two patients is therefore reassuring, although no conclusion can be drawn regarding tolerability in a broader nononcologic population. Within the limitations of this small uncontrolled study, WT1-DC vaccination was administered after anti-VEGF loading therapy without apparent vaccine-related safety concerns. However, the present data are insufficient to determine how WT1-DC vaccination should be integrated with existing anti-VEGF regimens in clinical practice. Nevertheless, given the small number of patients and the limited duration of follow-up, larger controlled studies with extended observations will be essential to confirm its long-term safety and to determine whether cumulative or delayed immune-related adverse events may occur with prolonged administration.

Patient AMD-WT1-01 experienced recurrent SRF during follow-up, requiring three additional aflibercept injections. In patient AMD-WT1-01, the BCVA decreased modestly over time; however, disease activity remained partially controlled, with visual acuity maintained at 0.4 at 88 weeks after therapy. In contrast, patient AMD-WT1-02 experienced long-term disease quiescence and maintained visual acuity without the need for additional anti-VEGF injections. In this study, WT1-specific immune responses, as assessed by IFN-γ and TNF-α production by T cells, were increased in both patients following in vitro stimulation with a novel WT1 peptide cocktail. However, the cytokine levels in the two patients returned to baseline levels within one month after the completion of the vaccination series. Because both patients received anti-VEGF loading therapy before vaccination and this study included only two heterogeneous cases without a control group, the reasons for the differing clinical courses remain uncertain. The more favorable course in patient AMD-WT1-02 cannot be distinguished from the effect of prior anti-VEGF therapy or from case-specific clinical factors, including diagnostic uncertainty regarding CNV etiology. Any discussion of a possible effect of the vaccination interval should therefore be regarded as speculative and hypothesis-generating only. Patient heterogeneity may also have contributed to the differing clinical courses. In particular, patient AMD-WT1-01 had the PCV subtype, which arises on a pachychoroid background and may differ biologically from typical nAMD [34]. However, given the absence of a control group and the extremely small and heterogeneous sample, no disease-specific inference can be made from the present study. These considerations underscore the importance of careful patient selection and cautious interpretation in future studies. Finally, the limitations of this study must be acknowledged. This report includes only two patients, and the natural history of nAMD is inherently heterogeneous. This pilot study was designed as a first-in-human exploratory investigation to evaluate the feasibility and safety of WT1-DC vaccination in nAMD; therefore, only two patients were enrolled. Accordingly, the extent to which the observed clinical course can be attributed to WT1-DC vaccination rather than baseline variability remains uncertain. Most importantly, the diagnostic classification of patient AMD-WT1-02 remains uncertain. Although this case was clinically managed as type 1 MNV within the nAMD spectrum on the basis of multimodal imaging and clinical course, the spherical equivalent was − 6.5 diopters, the axial length was 27.9 mm, and myopic CNV could not be excluded. Because this was the only case with a favorable long-term clinical course, this diagnostic uncertainty materially limits disease-specific interpretation of the present findings. Accordingly, the current data should not be interpreted as evidence of a specific therapeutic effect on nAMD.

Larger controlled studies incorporating biomarker analyses and extended follow-up will be essential to clarify whether WT1-DC vaccination can provide consistent and clinically meaningful benefits.

We next evaluated the induction of WT1-specific immune responses in these two patients. WT1-DTH responses remained undetectable throughout the treatment course. Moreover, in the WT1 tetramer assay of patient AMD-WT1-02 with HLA-A*24:02, WT1-specific CD8 + T cells restricted to HLA-A*24:02 were not detected in this study. Nevertheless, WT1 peptide-stimulated PBMCs from both patients exhibited functional WT1-specific CD4 + and CD8 + T-cell responses, resulting in the production of IFN-γ and TNF-α in vitro. In our previous clinical trial involving patients with WT1-expressing cancers, those who responded to treatment showed a positive WT1-DTH response and WT1-tetramer-positive CD8 + T cells following WT1-DC administration [27]. Compared with nAMD patients, cancer patients typically harbor a greater tumor burden of WT1-expressing cells, which may facilitate the spontaneous development of WT1-specific T cells in vivo.

Accordingly, the induction of WT1-specific immunity, as measured by the WT1-DTH test or WT1-tetramer assay, may be more difficult to detect in nAMD than in cancer. Importantly, in both nAMD patients, the production of IFN-γ and TNF-α by CD4 + and CD8 + T cells increased during WT1-DC vaccination upon stimulation with a novel WT1 peptide cocktail in vitro. Cytokine production in both patients returned to baseline levels within one month of completing the vaccination series. WT1-DTH responses remained negative in both patients, and WT1-tetramer–positive CD8 + T cells were not detected in the evaluable patient, indicating that robust in vivo systemic immunogenicity was not demonstrated in this study. The observed cytokine responses were obtained after in vitro peptide stimulation of PBMCs and should therefore be interpreted as exploratory laboratory findings rather than evidence of clinically meaningful immune activation in vivo. Although these findings may help inform future immunomonitoring strategies, no conclusion can be drawn regarding the relationship between these in vitro signals and clinical outcomes.

## Conclusions

To our knowledge, this study represents the first clinical evaluation of WT1-DC vaccination in patients clinically managed within the nAMD spectrum. In this two-patient pilot study, WT1-DC vaccination was feasible and well tolerated, with no vaccine-related toxicity observed. However, given the prior anti-VEGF loading therapy in both patients, the substantial difference in clinical course between the two heterogeneous cases, and the diagnostic uncertainty regarding CNV etiology in one patient, no conclusion can be drawn regarding therapeutic efficacy in nAMD. Likewise, although in vitro WT1 peptide–responsive T-cell activity was observed, clinically meaningful in vivo immunogenicity was not established. These findings should therefore be interpreted as only preliminary, descriptive, and hypothesis-generating.

## Electronic Supplementary Material

Below is the link to the electronic supplementary material.

Supplementary Material 1: Additional file 1. Longitudinal WT1-specific CD8+ T-cell responses to WT1-DC vaccination. WT1-specific CD8+ T-cell populations in patient AMD-WT1-02 with an HLA-A*24:02 allele were analyzed at predefined time points: before therapy, after the 15th vaccination, and 6 months after the final vaccination. WT1 tetramer–positive CD8+ T cells (left) and HIV tetramer–positive CD8+ T cells (right) are shown.

## Data Availability

The data that support the findings of this study are available from the corresponding author upon reasonable request.

## Abbreviations

AMD: Age-related macular degeneration
BCVA: Best corrected visual acuity
BVN: Branching vascular network
CNV: Choroidal neovascularization
CRT: Central retinal thickness
CTCAE: Common Terminology Criteria for Adverse Events
CTL: Cytotoxic T lymphocyte
DC: Dendritic cell
DTH: Delayed-type hypersensitivity
GM-CSF: Granulocyte-macrophage colony-stimulating factor
HLA: Human leukocyte antigen
IFN-γ: Interferon gamma
IL: Interleukin
IRF: Intraretinal fluid
KE: Klinische Einheit
MNV: Macular neovascularization
nAMD: Neovascular age-related macular degeneration
OCT: Optical coherence tomography
OCTA: Optical coherence tomography angiography
ORCC: Outer retina to choriocapillaris
PBMC: Peripheral blood mononuclear cell
PCV: Polypoidal choroidal vasculopathy
PRN: Pro re nata
SRF: Subretinal fluid
TAE: Treat-and-extend
TMV: Total macular volume
TNF-α: Tumor necrosis factor alpha
VEGF: Vascular endothelial growth factor
WT1: Wilms’ tumor 1
WT1-DC: WT1-pulsed dendritic cell
