# Supplementary material for: WT1-pulsed dendritic cell vaccination for neovascular age-related macular degeneration: a phase I pilot feasibility study
Source: BMC Ophthalmol. 2026 May 23;26:417. doi: 10.1186/s12886-026-04946-y (PMC13383492; doi:10.1186/s12886-026-04946-y)

Additional file 1. Longitudinal WT1-specific CD8 + T-cell responses to WT1-DC vaccination. WT1-specific CD8 + T-cell populations in patient AMD-WT1-02 with an HLA-A*24:02 allele were analyzed at predefined time points: before therapy, after the 15th vaccination, and 6 months after the final vaccination. WT1 tetramer–positive CD8 + T cells (left) and HIV tetramer–positive CD8 + T cells (right) are shown.


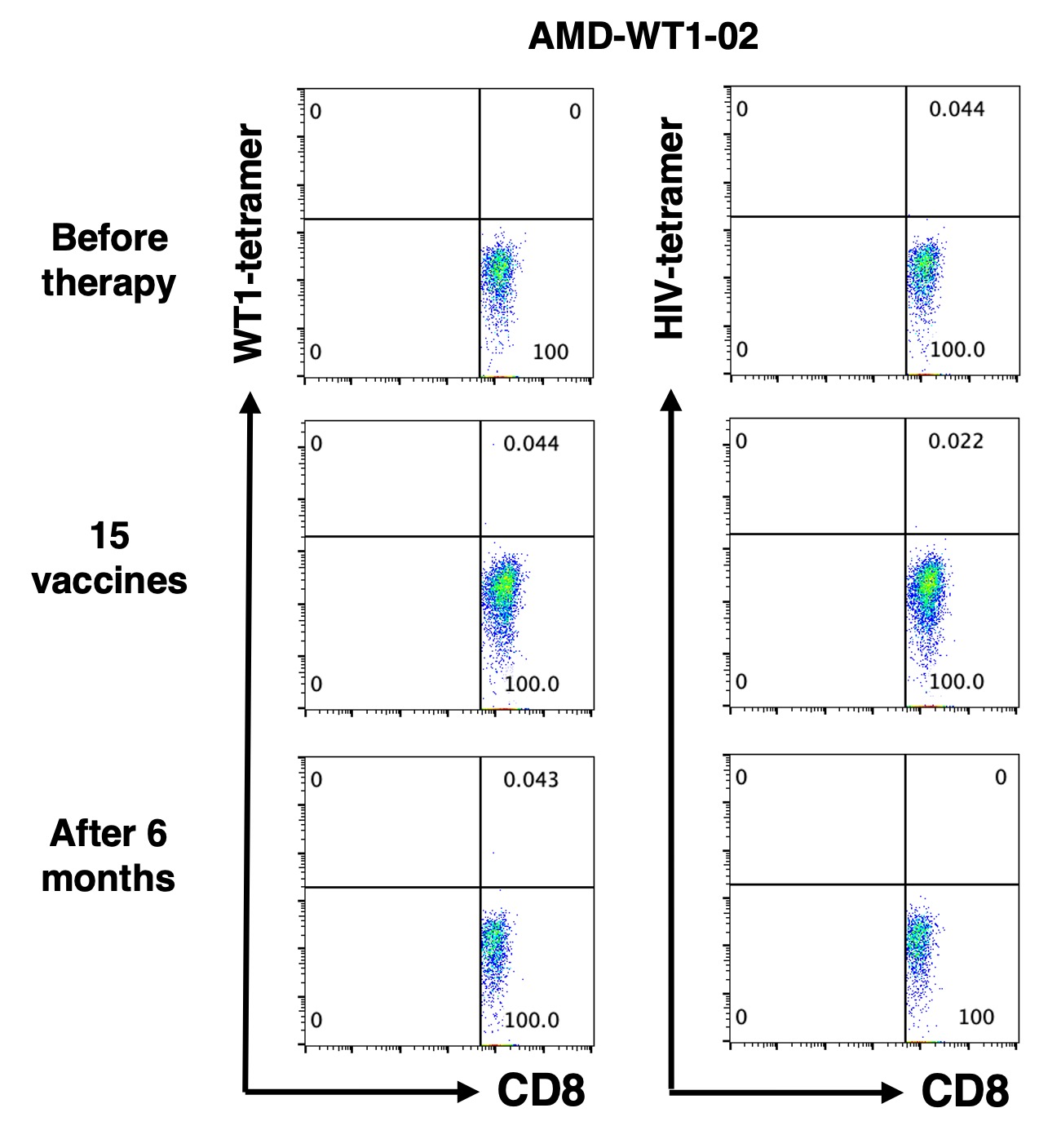

Supplement: Supplementary file 1 — Supplementary Material 1: Additional file 1. Longitudinal WT1-specific CD8+ T-cell responses to WT1-DC vaccination. WT1-specific CD8+ T-cell populations in patient AMD-WT1-02 with an HLA-A*24:02 allele were analyzed at predefined time points: before therapy, after the 15th vaccination, and 6 months after the final vaccination. WT1 tetramer–positive CD8+ T cells (left) and HIV tetramer–positive CD8+ T cells (right) are shown. [file 12886_2026_4946_MOESM1_ESM.docx]
